# Rates of Bronchopulmonary Dysplasia Following Implementation of a Novel Prevention Bundle

**DOI:** 10.1001/jamanetworkopen.2021.14140

**Published:** 2021-06-28

**Authors:** Maria Fe B. Villosis, Karine Barseghyan, Ma. Teresa Ambat, Kambiz K. Rezaie, David Braun

**Affiliations:** 1 Department of Pediatrics (Neonatology), Kaiser Permanente Panorama City, Panorama City, California; 2 Department of Research and Evaluation, Kaiser Permanente Southern California, Pasadena

## Abstract

**Importance:**

Bronchopulmonary dysplasia (BPD) rates in the United States remain high and have changed little in the last decade.

**Objective:**

To develop a consistent BPD prevention bundle in a systematic approach to decrease BPD.

**Design, Setting, and Participants:**

This quality improvement study included 484 infants with birth weights from 501 to 1500 g admitted to a level 3 neonatal intensive care unit in the Kaiser Permanente Southern California system from 2009 through 2019. The study period was divided into 3 periods: 1, baseline (2009); 2, initial changes based on ongoing cycles of Plan-Do-Study-Act (2010-2014); and 3, full implementation of successive Plan-Do-Study-Act results (2015-2019).

**Interventions:**

A BPD prevention system of care bundle evolved with a shared mental model that BPD is avoidable.

**Main Outcomes and Measures:**

The primary outcome was BPD in infants with less than 33 weeks’ gestational age (hereafter referred to as BPD <33). Other measures included adjusted BPD <33, BPD severity grade, and adjusted median postmenstrual age (PMA) at hospital discharge. Balancing measures were adjusted mortality and adjusted mortality or specified morbidities.

**Results:**

The study population included 484 infants with a mean (SD) birth weight of 1070 (277) g; a mean (SD) gestational age of 28.6 (2.9) weeks; 252 female infants (52.1%); and 61 Black infants (12.6%). During the 3 study periods, BPD <33 decreased from 9 of 29 patients (31.0%) to 3 of 184 patients (1.6%) (*P* < .001 for trend); special cause variation was observed. The standardized morbidity ratio for the adjusted BPD <33 decreased from 1.2 (95% CI, 0.7-1.9) in 2009 to 0.4 (95% CI, 0.2-0.8) in 2019. The rates of combined grades 1, 2, and 3 BPD decreased from 7 of 29 patients (24.1%) to 17 of 183 patients (9.3%) (*P* < .008 for trend). Grade 2 BPD rates decreased from 3 of 29 patients (10.3%) to 5 of 183 patients (2.7%) (*P* = .02 for trend). Adjusted median PMA at home discharge decreased by 2 weeks, from 38.2 (95% CI, 37.3-39.1) weeks in 2009 to 36.8 (95% CI, 36.6-37.1) weeks during the last 3 years (2017-2019) of the full implementation period. Adjusted mortality was unchanged, whereas adjusted mortality or specified morbidities decreased significantly.

**Conclusions and Relevance:**

A sustained low rate of BPD was observed in infants after the implementation of a detailed BPD system of care.

## Key Points

**Question:**

Is it possible to develop a consistent prevention bundle to decrease rates of bronchopulmonary dysplasia (BPD)?

**Findings:**

In this quality improvement study evaluating 484 infants with birth weights 501 to 1500 g, BPD in infants with less than 33 weeks’ gestational age decreased from 31% to 2%, and the adjusted standardized morbidity ratio among these infants decreased from 1.2 in 2009 to 0.4 in 2019. Adjusted median postmenstrual age at home discharge decreased by 2 weeks and adjusted mortality was unchanged, whereas adjusted mortality or specified morbidities decreased significantly.

**Meaning:**

A sustained low rate of BPD was observed after the implementation of a detailed BPD system of care.

## Teaser

This quality improvement study assesses whether a consistent, systematic prevention system of care decreases the rate of bronchopulmonary dysplasia in very low-birth-weight infants with gestational age less than 33 weeks.

## Introduction

Bronchopulmonary dysplasia (BPD) or chronic lung disease is a common, serious complication of prematurity.^1,2,3,4,5^ The incidence of BPD remains high and has been mostly unchanged during the last decade, ranging from 20% in California^6^ to 28% across the US,^7,8^ and 42% among infants less than 28 weeks’ gestation.^9^

Various interventions for the prevention of BPD have been studied^5^ although their individual effects on BPD rates have either been modest or have influenced only short-term benefits. Significant variation in risk-adjusted rates of BPD^6,10,11^ holds out the hope that there are existing care practice interventions that, if identified and propagated, could significantly decrease BPD rates. Lee et al^6^ estimated that achievement of top quartile rates of BPD in California would decrease the rate of BPD by 25%.

Some centers have reported consistently low and sustained rates of BPD,^12,13^ but efforts to identify key practice differences^14^ or to propagate their success through replicating some of their practices, such as bubble continuous positive airway pressure (CPAP), or efforts to avoid intubation have yielded little success, raising the concern that propagation of local successes^12^ or BPD prevention^15^ may not be possible.

The limited effect of individual interventions, wide variation in outcomes, and difficulty in propagating individual centers’ success suggest that BPD prevention is a system problem involving many types of management decisions and many individuals. The system has a better chance to succeed if the mental model of the care team is shared (ie, a concept based on a finding by Wu^16^ that patient safety may be improved through a consistent shared vision and implementation of care), if the management decision points are identified, and if execution of these management decisions is more consistent.^17^ The present report describes a single-center quality improvement initiative to develop a BPD prevention system of care that is associated with a decrease in rates of BPD.

## Methods

### Our Context and Study Population

Kaiser Permanente Panorama City (KPPC) has a 24-bed neonatal intensive care unit (NICU) and is part of Kaiser Permanente Southern California (KPSC), an integrated health care system with 4.7 million members and approximately 41 000 yearly births during the study period.^18^ All KPSC medical records are in 1 electronic system. Our NICU at KPPC has been a nonsurgical level 3 NICU since mid-2008 and became certified as a California Children’s Services community NICU in 2013. The annual local and referral birth population is approximately 4000 patients, including 300 NICU admissions of which approximately 45 neonates have birth weights (BWs) of 501 to 1500 g. Patients requiring surgical interventions are referred to KPSC surgical NICUs both prenatally and postnatally. Infants are transferred to lower acuity KPSC NICUs when they reach postmenstrual age (PMA) older than 32 weeks, require lower acuity respiratory support, such as high-flow nasal cannula at a fraction of inspired oxygen of 0.21, and are tolerating full feedings. Our team of 6 board-certified neonatologists (including all authors) provides in-house coverage 24 hours a day, 7 days a week. Daytime coverage is augmented by 3 board-certified pediatricians functioning as neonatal hospitalists. The same team of physicians provides in-house coverage 24 hours a day, 7 days a week at Kaiser Permanente Woodland Hills, the level 2 NICU where most of our patients with nonacute conditions are transported. This study followed the Standards for Quality Improvement Reporting Excellence (SQUIRE) reporting guideline for quality improvement studies. This study was approved by the KPSC Regional institutional review board with exemption of the requirement to obtain informed consent because the data were deidentified. No one received compensation or was offered any incentive for participating in this study.

This study was started as a response to an increase in rates of BPD in 2009, the first full year after moving our level 3 NICU to a different medical center with associated changes in physician, nursing, and respiratory therapist staff. The quality improvement team included neonatologists (all authors), respiratory therapists, NICU registered nurses, and a multidisciplinary team. The aim of the study was to decrease BPD rates in our NICU through Plan-Do-Study-Act cycles between 2010 and 2019. All inborn and outborn NICU admissions with BWs of 501 to 1500 g from 2009 to 2019 were included in the study.

### Process

In 2009, the quality improvement team at our NICU agreed that our increased BPD rate and inconsistencies in pulmonary management had to be improved. We agreed to work together to increase the consistency and quality of respiratory care–related practices. The rate of BPD was the major outcome measure. Our quality improvement team’s initial assessment was that a relative lack of a shared mental model for managing respiratory care and the related inconsistency of care were important factors associated with our worsening BPD rates.

### Key Drivers

During the study period, we developed a list of key drivers for BPD prevention (Table 1). The key drivers included (1) a shared mental model that prevention of BPD is possible; (2) prevention vs rescue therapy to support postnatal lung growth and to minimize the inflammatory cascade and oxygen toxicity that lead to BPD; (3) consistent management across the team to minimize variations in care; and (4) management decision points based on developmental stages of the lung.

**Table 1.** Key Drivers

Driver	Initial changes period response (2010-2014)	Full implementation period response (2015-2019)
Shared mental model that BPD is avoidable but requires aggressive preventative care Consistent management practices optimize the chance for success	Discussion on daily rounds of compliance with protocol for each infant Discussion at weekly neonatology meetings of challenges and opportunities regarding care agreements Changes in management made only after consensus of neonatologists	Written agreements on respiratory care specifics
Postnatal age– and PMA-specific interventions	Delivery room: CPAP at a minimum Low threshold for intubation and surfactant use Pressure support ventilation with volume guarantee for invasive ventilation High-frequency oscillatory ventilation for rescue Administration of at least 1 surfactant dose if on ventilator Treat agitation through improved respiratory support or nonpharmacological comforting; sedation used infrequently Criteria for extubation Postextubation: nasal CPAP or nasal intermittent mandatory ventilation phasing into NAVA (2012) Wean to high-flow nasal cannula no earlier than 32 wk PMA RAM Cannula (Neotech) nasal interface from 2012 for NAVA and nasal CPAP Vapotherm (Exeter) for high-flow nasal cannula from 2014 Dexamethasone rescue criteria and protocol Prophylactic caffeine until 34 wk PMA; epoetin from full feedings to discharge; vitamin A for 4 wk,^a^ intravenous immunoglobulin until 1250 g^a^ Rescue budesonide and albuterol^a^ Feeding protocol including breast milk as base for feedings (bovine supplements) Fluid restriction with avoidance of diuretics Ureaplasma and mycoplasma screening cultures and presumptive azithromycin pending results of cultures Antibiotics only for symptoms and continued only if positive blood, urine, or cerebrospinal fluid cultures	Written, more explicit “No BPD Roadmap” implemented especially for gestational age less than 28 wk and birth weight less than 1000 g, including high-frequency oscillatory ventilation as primary invasive ventilation,^a^ noninvasive ventilation as NAVA until 32 wk, human milk for both base and fortifiers Expanded criteria for surfactant use Observation rather than treatment of PDA Avoid pharmacologic treatment of reflux

Abbreviations: BPD, bronchopulmonary dysplasia; CPAP, continuous positive airway pressure; NAVA, neurally adjusted ventilatory assist; PDA, patent ductus arteriosus; PMA, postmenstrual age.

^a^ Gestational age less than 28 weeks or birth weight less than 1000 g.

### Implementation

The evolution of our care practices in response to the key drivers is given in Table 1. After a 1-year baseline period (2009), we entered a period of initial changes involving a range of respiratory and nonrespiratory interventions. The implementation included discussion of our system of care for each infant at daily sign-out rounds and debriefs on the process at weekly neonatology meetings. Changes in care practices were made if a new consensus was reached. Compliance with the consensus guideline was discussed at daily sign-out rounds and debriefs. Initial consensus practices included volume-targeted or high-frequency ventilation modalities for intubated patients, group decisions regarding rescue care with the administration of dexamethasone, postextubation pathway, prophylactic caffeine, vitamin A, and surfactant therapy.

By the full implementation period, the group’s belief in the value of the shared mental model decision point–specific consensus had grown such that more detailed respiratory and nonrespiratory management protocols were adopted and circulated as written protocols addressing postnatal and postconceptional age-specific respiratory management. Preventing BPD was envisioned as protecting against lung injury and supporting lung growth. Emphasis was placed on proactive intervention to prevent deterioration rather delaying intervention until deterioration occurred and rescue care was required. High expectations were embraced for acceptable respiratory status with an emphasis on full alveolar recruitment. The system of care in the full implementation period is described in eFigures 1, 2, and 3 and eAppendix 2 in the Supplement. We appreciate that studies of these interventions have shown variable benefit individually. Our intent was to assess the net association of their adoption in successive Plan-Do-Study-Act cycles with the outcome.

### Source of Data for Analysis

Data sources included KPPC data that were submitted to the Vermont Oxford Network (VON), California Perinatal Quality Care Collaborative (CPQCC), and KPSC electronic medical records. Unless otherwise indicated, the data were obtained from VON.

### Data Elements for Analysis

Maternal and infant demographic characteristics and respiratory care practice measures available in the VON registry for the KPPC NICU were collected for each period. The primary outcome was “BPD <33” as defined by VON to be infants younger than 33 weeks’ gestational age (GA) at birth and at our center on supplemental oxygen at 36 weeks’ PMA or, if discharged home before 36 weeks’ PMA, on supplemental oxygen at discharge.

Secondary BPD outcomes included the following. To address potential case mix bias, we reported our center’s unadjusted BPD rates for 2 subgroups composed of infants with GA less than 28 weeks or less than 26 weeks and reported our center’s adjusted BPD <33 as calculated by VON. To address potential BPD case definition bias, we used the following grading system that uses level of respiratory support administered at 36 weeks’ PMA regardless of prior or current oxygen therapy to define disease severity in infants of GA less than 33 weeks: grade 1, nasal cannula airflow 2 liters per minute or lower; grade 2, nasal cannula airflow higher than 2 liters per minute or noninvasive positive airway pressure; and grade 3, invasive mechanical ventilation.^18^ These diagnostic criteria are reported to best predict death or respiratory morbidity through 18 to 26 months’ corrected age. We also evaluated the use of supplemental oxygen or tracheostomy at discharge to home.

The NICU length of stay as another measure of BPD severity was presented as risk-adjusted PMA at discharge for our center reported by CPQCC (data accessed November 2020) for patients with 22 to 29 weeks’ GA or BW 401 to 1500 g. This population is similar but not identical to the population with BW 501 to 1500 g. The metric adjusts for the following covariates: GA, small for GA, malformation, multiple gestation, 5-minute Apgar score, sex, outborn, and no prenatal care. Balancing measures were VON measures of adjusted mortality and adjusted mortality or specified morbidities (eAppendix 1 in the Supplement).

### Statistical Analysis

**Figure 1.**
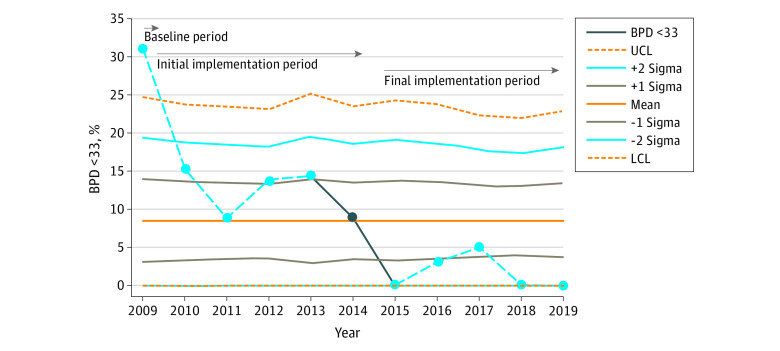
P-chart Displaying Bronchopulmonary (BPD) Dysplasia Incidence in Infants With Birth Weights of 501 to 1500 g and Less Than 33 Weeks’ Gestation BPD <33 indicates BPD in patients of gestational age less than 33 weeks; LCL, lower control limit; and UCL, upper control limit. The dashed light blue–solid dark blue line indicates BPD less than 33, and the LCL is 0%.

Statistical process control charts (QI Macros; KnowWare International Inc) were used to display and analyze data for unadjusted BPD <33 over time. Special cause variation—an unexpected variation that results from unusual occurrences—was based on Montgomery rules,^19^ and the results are presented as a p-chart (Figure 1).

Adjusted rates of BPD <33, mortality, and mortality or specified morbidities were obtained from VON reported as a shrunken standardized morbidity ratio (SMR), which is the term VON uses to describe the risk-adjusted outcomes calculated for each hospital in the registry. The SMR includes patient-level adjustments for selected risk factors: GA, birth weight, small for GA, severity of congenital anomaly, multiple gestation, 1-minute Apgar score, sex, vaginal delivery, birth location (inborn or outborn), and altitude of center.

Unadjusted demographic characteristics, care practice, and outcome measures between the 3 study periods were compared by the χ^2^ test or the Fischer exact test for categorical variables and by the Kruskal-Wallis test for continuous variables. Trends across the study periods were evaluated by the Cochran-Armitage test if the overall test was significant. A 2-sided *P* < .05 was considered statistically significant. Statistical analyses were performed using SAS, version 9.4 software (SAS Institute Inc).

## Results

### Demographic Characteristics

There were 484 infants with BW of 501 to 1500 g admitted during the study period, of whom 435 (89.9%) were inborn, 232 were male (47.9%), 252 were female (52.1%), and 61 were Black (12.6%) infants (Table 2). The mean (SD) BW of the population was 1070 (277) g, and the mean (SD) GA was 28.6 (2.9) weeks, with 190 infants (39%) born at GA less than 28 weeks. During the study period, the rates of GA less than 28 weeks and GA less than 26 weeks increased, rates of Black patients decreased, and 476 of 484 mothers (98.3%) received prenatal care.

**Table 2.** Demographic Characteristics and Care Practice Measures and Outcomes

Measure	No./total No. (%)				*P* value	
	All years (2009-2019)	Baseline (2009)	Initial changes (2010-2014)	Final implementation (2015-2019)	χ^2^ or Fisher exact test^a^	Cochran- Armitage test
**Demographic characteristics**						
Births and admissions, No.	484	45	216	223		
Birth weight, mean (SD), g	1069.93 (277.39)	1069.93 (277.39)	1095.99 (276.22)	1045.89 (281.06)	.17^b^	NA
GA, mean (SD), wk	28.61 (2.88)	28.75 (2.33)	28.88 (2.73)	28.35 (3.04)	.18^b^	NA
GA <33 wk	449/484 (92.8)	43/45 (95.6)	201/216 (93.1)	205/223 (91.9)	.76^a^	NA
GA <28 wk	190/484 (39.3)	14/45 (31.1)	75/216 (34.7)	101/223 (45.3)	.04^a^	.01
GA <26 wk	100/484 (20.7)	5/45 (11.1)	37/216 (17.1)	58/223 (26.0)	.02^a^	.005
SGA	83/484 (17.1)	8/45 (17.8)	33/216 (15.3)	42/223 (18.8)	.61	NA
Male	232/484 (47.9)	24/45 (53.3)	104/216 (48.1)	104/223 (46.6)	.72	NA
Female	252/484 (52.1)	21/45 (46.7)	112/216 (51.9)	119/223 (53.4)		
Multiple gestation	157/484 (32.4)	16/45 (35.6)	66/216 (30.6)	75/223 (33.6)	.71	NA
Race/ethnicity						
Asian	51/484 (10.5)	5/45 (11.1)	20/216 (9.3)	26/223 (11.7)	.71	NA
Black	61/484 (12.6)	10/45 (22.2)	30/216 (13.9)	21/223 (9.4)	.05^a^	.02
Hispanic	216/484 (44.6)	18/45 (40.0)	98/216 (45.4)	100/223 (44.8)	.80	NA
Native American	0/484 (0)	0/45	0/216	0/223	NA	NA
Other	12/484 (2.5)	0/45	8/216 (3.7)	4/223 (1.8)	.33^a^	NA
White	144/484 (29.8)	12/45 (26.7)	60/216 (27.8)	72/223 (32.3)	.52	NA
Prenatal care	476/484 (98.3)	43/45 (95.6)	214/216 (99.1)	219/223 (98.2)	.20^a^	NA
Inborn	435/484 (89.9)	42/45 (93.3)	197/216 (91.2)	196/223 (87.9)	.43^a^	NA
Cesarean delivery	339/484 (70.0)	32/45 (71.1)	155/216 (71.8)	152/223 (68.2)	.70	NA
1-min Apgar score, mean (SD)	6.16 (2.26)	6.27 (2.24)	6.12 (2.25)	6.18 (2.27)	.97^b^	NA
5-min Apgar score, mean (SD)	7.73 (2.01)	7.73 (2.19)	7.61 (2.2)	7.83 (1.78)	.66^b^	NA
Major anomaly	16/484 (3.3)	1/45 (2.2)	6/216 (2.8)	9/223 (4.0)	.70^a^	NA
**Care practices**						
Antenatal steroid	444/484 (91.7)	42/45 (93.3)	199/216 (92.1)	203/223 (91.0)	.88^a^	NA
Initial resuscitation with ETT ventilation	325/484 (67.1)	35/45 (77.8)	142/216 (65.7)	148/223 (66.4)	.32	NA
Initial resuscitation with surfactant	246/482 (51.0)	25/45 (55.6)	85/214 (39.7)	136/223 (61.0)	<.001	NA
High-flow nasal cannula after initial resuscitation	350/465 (75.3)	31/45 (68.9)	155/204 (76.0)	164/216 (75.9)	.53^a^	NA
Nasal CPAP after initial resuscitation	369/465 (79.4)	25/45 (55.6)	158/204 (77.5)	186/216 (86.1)	<.001	*<*.001
Nasal ventilation after initial resuscitation	296/465 (63.7)	16/45 (35.6)	106/204 (52.0)	174/216 (80.6)	<.001	<.001
Conventional ventilation after initial resuscitation	360/465 (77.4)	37/45 (82.2)	163/204 (79.9)	160/216 (74.1)	.27	NA
High-frequency ventilation after initial resuscitation	155/465 (33.3)	16/45 (35.6)	29/204 (14.2)	110/216 (50.9)	<.001	NA
Surfactant at any time	364/484 (75.2)	37/45 (82.2)	158/216 (73.1)	169/223 (75.8)	.46	NA
Steroids for BPD at any site	58/464 (12.5)	10/45 (22.2)	21/203 (10.3)	27/216 (12.5)	.08	NA
Nitric oxide	6/484 (1.2)	0/45 (0)	2/216 (0.9)	4/223 (1.8)	.82^a^	NA
Indomethacin for any reason	86/465 (18.5)	16/45 (35.6)	63/204 (30.9)	7/216 (3.2)	<.001	<.001
Transferred to another facility	118/465 (25.4)	25/45 (55.6)	46/204 (22.5)	47/216 (21.8)	<.001	<.001
Acute transfer	38/465 (8.2)	5/45 (11.1)	19/204 (9.3)	14/216 (6.5)	0.51	NA
Nonacute transfer to Woodland Hills	51/465 (11.0)	11/45 (24.4)	15/204 (7.4)	25/216 (11.6)	<.001	NA
Nonacute transfer to other site	29/465 (6.2)	9/45 (20.0)	12/204 (5.9)	8/216 (3.7)	<.003	NA
**GA <33 wk excluded from BPD <33 measure**						
Died before 36 0/7 wk PMA including DR deaths	47/449 (10.5)	5/43 (11.6)	22/201 (10.9)	20/205 (9.8)	.88	NA
Alive but transferred to centers with data not linked by VON to our center	24/402 (6.0)	9/38 (23.7)	14/179 (7.8)	1/185 (0.5)	<.001^a^	<.001
**Major BPD-related outcomes in GA <33**						
BPD <33 wk (VON)	32/378 (8.5)	9/29 (31.0)	20/165 (12.1)	3/184 (1.6)	<.001^a^	<.001
Grade 1 BPD	29/377 (7.7)	4/29 (13.8)	14/165 (8.5)	11/183 (6.0)	.26^a^	.14
Grade 2 BPD	21/377 (5.6)	3/29 (10.3)	13/165 (7.9)	5/183 (2.7)	.13^a^	.02
Grade 3 BPD	2/377 (0.5)	0/29 (0)	1/165 (0.6)	1/183 (0.5)	>.99	NA
Any grade BPD	52/377 (13.8)	7/29 (24.1)	28/165 (17.0)	17/183 (9.3)	.03	<.008
Other outcomes						
Tracheostomy at discharge home in GA <33 wk	1/376 (0.3)	0/29 (0)	0/164 (0)	1/183 (0.5)	.59	NA
Oxygen at 28 d in >33 wk GA	85/377 (22.5)	11/29 (37.9)	45/165 (27.3)	29/183 (15.8)	<.003	NA
BPD <28 wk (VON)	14/135 (10.4)	4/8 (50.0)	9/46 (19.6)	1/81 (1.2)	<.001^a^	<.001
BPD <26 wk (VON)	6/53 (11.3)	0/0 (0)	5/14 (35.7)	1/39 (2.6)	<.004	NA
Oxygen at discharge home in GA <33 wk	18/377 (4.8)	4/29 (13.8)	10/165 (6.1)	4/183 (2.2)	.03^a^	.01
Pneumothorax at any site	15/465 (3.2)	4/45 (8.9)	5/204 (2.5)	6/216 (2.8)	.10^a^	NA
PDA	140/463 (30.2)	18/45 (40.0)	62/202 (30.7)	60/216 (27.8)	.22	NA
PDA ligation	11/484 (2.3)	0/45 (0)	6/216 (2.8)	5/223 (2.2)	.66^a^	NA
Enteral feeding at discharge with any human milk	309/336 (92.0)	16/17 (94.1)	141/156 (90.4)	152/163 (93.3)	.70^a^	NA
Any PIH at any location	65/442 (14.7)	3/36 (8.3)	25/193 (13.0)	37/213 (17.4)	.28^a^	NA
Severe IVH	21/442 (4.8)	1/36 (2.8)	9/193 (4.7)	11/213 (5.2)	>.99^a^	NA
Cystic PVL	1/442 (0.2)	0/36	1/193 (0.5)	0/213	.52^a^	NA
NEC at any site	8/464 (1.7)	3/45 (6.7)	2/203 (1.0)	3/216 (1.4)	.045^a^	.10
Nosocomial infection at any site	10/448 (2.2)	3/40 (7.5)	4/198 (2.0)	3/210 (1.4)	.07^a^	NA
Severe ROP	11/372 (3.0)	1/28 (3.6)	6/156 (3.8)	4/188 (2.1)	.54^a^	NA
Extreme LOS (survivors)	4/435 (0.9)	0/40	3/192 (1.6)	1/203 (0.5)	.56^a^	NA
Death or morbidity	102/482 (21.2)	17/45 (37.8)	50/214 (23.4)	35/223 (15.7)	<.002	<.001

Abbreviations: BPD, lung disease bronchopulmonary dysplasia; CPAP, continuous positive airway pressure; DR, delivery room; ETT, endotracheal tube; GA, gestational age; LOS, length of hospital stay; NA, not applicable; NEC, stage 2 or higher necrotizing enterocolitis; PMA, postmenstrual age; PDA, patent ductus arteriosus; PIH, periventricular-intraventricular hemorrhage; PVL, periventricular leukomalacia; severe IVH, grade 3 or 4 periventricular-intraventricular hemorrhage; severe ROP, stage 3 or greater retinopathy of prematurity; SGA, small for gestational age; VON, Vermont Oxford Network.

^a^ Fisher exact test.

^b^ Kruskal-Wallis test.

### Care Practice Measures

During the study period, care practice measures (process measures) that increased significantly included high-frequency ventilation and noninvasive ventilation (Table 2). Process measures that decreased significantly included indomethacin administration and transfers. Acute transfers did not change significantly, with 38 of 465 patients (8.2%) throughout the study. Nonacute transfers to the Woodland Hills level 2 NICU included a total of 51 of 465 patients (11.0%). Nonacute transfers to other centers decreased from 9 of 45 patients (20.0%) to 18 of 216 patients (3.7%); this decrease was associated with California Children’s Services certification of the KPPC NICU. Process measures that did not change significantly included patent ductus arteriosus (PDA) ligation (11 of 484 patients [2.3%]) and administration of antenatal steroid (444 of 484 patients [91.7%]), surfactant (364 of 484 patients [75.2%]), postnatal steroid for BPD (58 of 464 patients [12.5%]), or nitric oxide (6 of 484 patients [1.2%]).

### Outcomes

**Figure 2.**
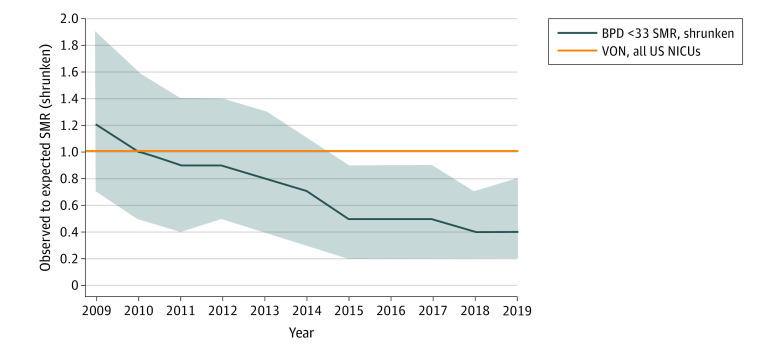
Risk-Adjusted Bronchopulmonary Dysplasia (BPD) in Infants With Birth Weights of 501 to 1500 g and Less than 33 Weeks’ Gestation BPD less than 33 indicates BPD in patients of gestational age less than 33 weeks; NICU, neonatal intensive care unit; SMR, standardized morbidity ratio; and VON, Vermont Oxford Network. Shaded area indicates 95% CIs.

Of the study population of 484 patients, 35 were GA 33 weeks or older and were excluded for the determination of BPD <33. There were 449 patients with GA less than 33 weeks, of whom 47 died before 36 weeks’ PMA (Table 2). Most of those deaths occurred in extremely preterm infants born at lower or lowest periviable GA range, with more than half of early deaths occurring in the delivery room or within the first hour of NICU admission. The BPD <33 status was missing or not included in the VON BPD <33 metric for 24 patients. Of the remaining 378 patients, the rate of BPD <33 decreased from 9 of 29 patients (31.0%) to 3 of 184 patients (1.6%) during the 3 study periods (*P* < .001), and special cause variation was shown by statistical process control methods (Figure 1). The rate of BPD for patients of GA less than 28 weeks decreased from 4 of 8 patients (50.0%) to 1 of 81 patients (1.2%) (*P* < .001). The rate of BPD for patients of GA less than 26 weeks was 1 of 39 patients (2.6%) in the final implementation period, but the decreasing trend was not significant as assessed by the Cochran-Armitage test. The SMR for the adjusted BPD <33 decreased from 1.2 (95% CI, 0.7-1.9) in the baseline period to 0.4 (95% CI, 0.2-0.8) in 2019 at the end of the full implementation period with a combined rate of 0.2 (95% CI, 0.1-0.4) from 2017 to 2019 (Figure 2).

Grades of BPD could be assigned only to 377 of 378 patients whose combined grades 1, 2, and 3—referred to as any grade BPD—decreased from 7 of 29 patients (24.1%) to 17 of 183 patients (9.3%) (*P* < .008 for trend). The rate of grade 1 BPD decreased from 4 of 29 patients (13.8%) to 11 of 183 patients (6.0%), but this decreasing trend was not significant as assessed by the Cochran-Armitage test, whereas the decrease in the rate of grade 2 BPD from 3 of 29 patients (10.3%) to 5 of 183 patients (2.7%) was significant (*P* = .02 for trend). Grade 3 BPD was low across the study period with only 2 patients, 1 patient each for the initial and final implementation period. Oxygen at home discharge decreased from 4 of 29 patients (13.8%) to 4 of 183 patients (2.2%) (*P* = .03). One infant required tracheostomy tube placement in the last period (Table 2).

**Figure 3.**
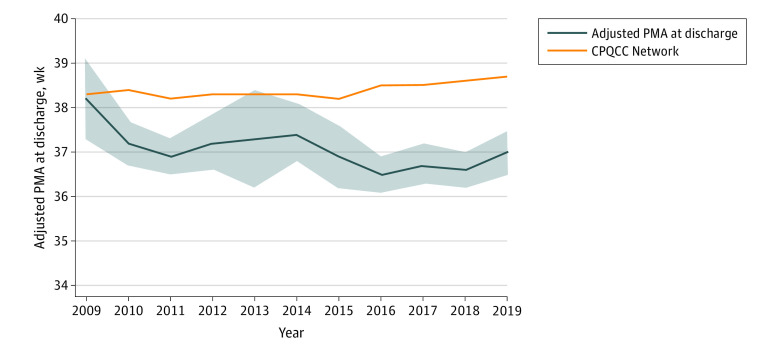
Risk-Adjusted Median Postmenstrual Age (PMA) at Home Discharge in Infants With Birth Weights of 401 to 1500 g or 22 to 29 Weeks’ Gestation CPQCC indicates California Perinatal Quality Care Collaborative network. Shaded area indicates 95% CIs.

Adjusted median PMA at hospital discharge (Figure 3) decreased from 38.2 weeks (95% CI, 37.3-39.1 weeks) in the baseline period (2009) to 37.0 weeks (95% CI, 36.5-37.5 weeks) in 2019 at the end of the full implementation period, and was 36.8 weeks (95% CI, 36.6-37.1 weeks) for combined years 2017 through 2019. Both of these values were significantly lower than all centers combined in the CPQCC registry. The adjusted mortality was unchanged during the study periods (eFigure 4 in the Supplement), whereas adjusted mortality or specified morbidities decreased significantly (eFigure 5 in the Supplement).

## Discussion

During a 10-year period, we observed a substantial, sustained decrease in the metric BPD <33, from 31.0% in 2009 to 1.6% in the most recent 5-year period. This decrease was unlikely due to case mix bias because the SMR for BPD less than 33 weeks decreased considerably, and BPD rates were also low in the unadjusted subgroups of GA less than 28 weeks and of GA less than 26 weeks. This decrease was also unlikely due to case ascertainment bias because we had a low rate of patients with missing BPD data: 5.0% overall and only 1 of 185 patients in the full implementation period. In addition, the decrease was unlikely due to case definition bias because BPD decreased significantly by all of the following definitions: BPD <33, any grade BPD, grade 2 BPD, and oxygen at discharge. Grade 1 BPD decreased but the trend was not significant, whereas grade 3 BPD remained low throughout the study. The adjusted median PMA at discharge to home associated with cost^20^ and long-term outcome^21,22^ also decreased significantly to 36.8 weeks for the combined years 2017 through 2019, which is 2 weeks less than the CPQCC median. The decrease in BPD <33 was not at the cost of an increase in balancing factors because adjusted mortality was stable and there was a significant decrease in adjusted mortality or specified morbidities, a measure associated with long-term outcomes.^23^

Given all these considerations, our outcomes were likely associated with our current care practices rather than with analytical biases. The reasons underpinning our success may be found in the evidence base for each individual intervention we adopted in our “BPD Prevention Bundle of Interventions” and “No BPD Roadmap.” The postnatal interventions of varying degrees of evidence supporting efficacy in preventing BPD included high-frequency ventilation^24^; volume-targeted ventilation^25^; surfactant therapy^26,27^; CPAP use^15,25,28,29,30,31^; administration of caffeine^32,33,34,35^; vitamin A^36,37,38^; azithromycin^39^; human breast milk^40^; and inhaled or systemic steroids^41,42,43,44,45^; and lowered oxygen saturation targets of 85% to 95% up to 34 weeks’ gestation.^46,47^ The interventions that we adopted that improved various aspects of short-term respiratory function, although they have not yet been shown to decrease BPD, included the use of extended CPAP,^48^ neurally adjusted ventilatory assist,^25,28,29,30,40^ high-flow nasal cannula,^49,50,51,52^ and inhaled β-agonists.^53^ Permissive hypercarbia and the use of diuretics were not encouraged in our bundle.^54,55^ Epoetin efficacy has been reported in studies but not in large randomized clinical trials or meta-analyses.^56,57,58,59^ Fluid restriction is included in our care practices, but its value in preventing or treating BPD has not been well established.^60,61^ Interventions we minimized that may increase BPD were treatment of PDA^62,63,64^ and antibiotic use.^65^

The decrease in BPD <33 associated with the changes we implemented highlights the importance of a more fully delimited and implemented system of care over individual interventions, that is, the whole was greater than the sum of its parts. Our favorable outcomes were associated with the expansion of our shared mental model of BPD prevention and the standardization of management for a range of postnatal age-specific and postconceptional age-specific clinical scenarios for which management was defined (eAppendix 2 and eFigures 1, 2, and 3 in the Supplement).

For the shared mental model, preventing BPD was envisioned as protecting against lung injury and supporting lung growth, with emphasis on proactive and optimized respiratory support to prevent deterioration rather than on rescue care. Focused respiratory care interventions aimed at avoiding alveolar de-recruitment and oxygen toxicity were central. Pneumonia, PDA, or reflux were rarely accepted as reasons for respiratory deterioration. This may have had the additional benefit of lowering PDA ligation rates and antibiotic use, which have been associated with increased BPD rates. The changes in our shared mental model and management were the product of a sustained quality improvement effort.

The detailed postnatal and PMA standardization of care may have been a factor in our improvement because standardized practice itself tends to improve outcomes in clinical settings, including ventilator care in NICUs.^17,66,67^ Our efforts to standardize practice, strong leadership, a consensus/commitment culture, daily bedside rounds, weekly meetings, and vignette case discussions were necessary to achieve these outcomes.

Regarding the system of care, the bundle of these interventions in its entirety was associated with our favorable outcomes although we do not know the relative contribution of each intervention. We recommend that the first focus of subsequent studies be on replicating our system of care and our outcomes rather than on dissecting each element of the bundle to find the minimally required components to achieve favorable outcomes.

### Strengths and Limitations

This study has several strengths. First, the degree of the observed decrease in BPD rates was clinically important and statistically significant. Second, the chance of case mix bias, case ascertainment bias, or case definition bias was unlikely. Third, we provided descriptions of our “No BPD Roadmap,” with ventilatory and nonventilatory management as clinical tools to facilitate replication of the implementation details of our strategy.

There are a few limitations to this study. This is a single-center study with a small sample size. The efficacy of this BPD prevention bundle has not been studied in a surgical population.

## Conclusions

We observed a substantial, sustained decrease in BPD rates in association with the development and implementation of a detailed BPD prevention bundle. Our success may be associated with a shared mental model of care that BPD is preventable, the details of the system of care, and the consistency of its execution. We believe the bundle of care described in this report is sufficiently detailed to enable researchers to assess whether these outcomes can be replicated at other centers.

## Notes

Supplement. **eAppendix 1.** Shrunken Standardized Morbidity Ratio **eFigure 1.** No BPD Road Map: Ventilatory Management for High-Risk Infants of GA <28 Weeks or BW <1000 g **eFigure 2.** No BPD Road Map: Ventilatory Management for High-Risk Infants of GA 28-31 Weeks and BW ≥1000 g **eFigure 3.** No BPD Road Map: Nonventilatory Management/Pharmacologic Therapies and Other Strategies for Very Low-Birth-Weight Infants (BW <1500 g) **eFigure 4.** Risk-Adjusted Mortality in Infants 501 to 1500 g **eFigure 5.** Risk-Adjusted Mortality or Specified Morbidities in Infants 501 to 1500 g **eAppendix 2.** BPD Prevention Bundle Implementation

## References

[1] Walsh MC, Morris BH, Wrage LA, ; National Institutes of Child Health and Human Development Neonatal Research Network. Extremely low birthweight neonates with protracted ventilation: mortality and 18-month neurodevelopmental outcomes. J Pediatr. 2005;146(6):798-804. doi:10.1016/j.jpeds.2005.01.047 15973322

[2] Schmidt B, Asztalos EV, Roberts RS, Robertson CM, Sauve RS, Whitfield MF; Trial of Indomethacin Prophylaxis in Preterms (TIPP) Investigators. Impact of bronchopulmonary dysplasia, brain injury, and severe retinopathy on the outcome of extremely low-birth-weight infants at 18 months: results from the trial of indomethacin prophylaxis in preterms. JAMA. 2003;289(9):1124-1129. doi:10.1001/jama.289.9.1124 12622582

[3] Natarajan G, Pappas A, Shankaran S, . Outcomes of extremely low birth weight infants with bronchopulmonary dysplasia: impact of the physiologic definition. Early Hum Dev. 2012;88(7):509-515. doi:10.1016/j.earlhumdev.2011.12.013 22236557 PMC3686277

[4] Mowitz ME, Ayyagari R, Gao W, Zhao J, Mangili A, Sarda SP. Health care burden of bronchopulmonary dysplasia among extremely preterm infants. Front Pediatr. 2019;7:510. doi:10.3389/fped.2019.00510 31921723 PMC6921371

[5] Higgins RD, Jobe AH, Koso-Thomas M, . Bronchopulmonary dysplasia: executive summary of a workshop. J Pediatr. 2018;197:300-308. doi:10.1016/j.jpeds.2018.01.043 29551318 PMC5970962

[6] Lee HC, Liu J, Profit J, Hintz SR, Gould JB. Survival without major morbidity among very low birth weight infants in California. Pediatrics. 2020;146(1):e20193865. doi:10.1542/peds.2019-3865 32554813 PMC7329260

[7] Vermont Oxford Network. Expanded database. March 25, 2020. Accessed September 7, 2020. https://public.vtoxford.org/data-and-reports/expanded-database

[8] Horbar JD, Edwards EM, Greenberg LT, . Variation in performance of neonatal intensive care units in the United States. JAMA Pediatr. 2017;171(3):e164396. doi:10.1001/jamapediatrics.2016.4396 28068438

[9] Stoll BJ, Hansen NI, Bell EF, ; Eunice Kennedy Shriver National Institute of Child Health and Human Development Neonatal Research Network. Neonatal outcomes of extremely preterm infants from the NICHD Neonatal Research Network. Pediatrics. 2010;126(3):443-456. doi:10.1542/peds.2009-2959 20732945 PMC2982806

[10] Siffel C, Kistler KD, Lewis JFM, Sarda SP. Global incidence of bronchopulmonary dysplasia among extremely preterm infants: a systematic literature review. J Matern Fetal Neonatal Med. 2021;34(11):1721-1731. doi:10.1080/14767058.2019.1646240 31397199

[11] Lapcharoensap W, Gage SC, Kan P, . Hospital variation and risk factors for bronchopulmonary dysplasia in a population-based cohort. JAMA Pediatr. 2015;169(2):e143676. doi:10.1001/jamapediatrics.2014.3676 25642906

[12] de Klerk AM, de Klerk RK. Use of continuous positive airway pressure in preterm infants: comments and experience from New Zealand. Pediatrics. 2001;108(3):761-763. doi:10.1542/peds.108.3.761 11533347

[13] Avery ME, Tooley WH, Keller JB, . Is chronic lung disease in low birth weight infants preventable? a survey of eight centers. Pediatrics. 1987;79(1):26-30. 3797169

[14] Van Marter LJ, Allred EN, Pagano M, . Do clinical markers of barotrauma and oxygen toxicity explain interhospital variation in rates of chronic lung disease? the Neonatology Committee for the Developmental Network. Pediatrics. 2000;105(6):1194-1201. doi:10.1542/peds.105.6.1194 10835057

[15] Wright CJ, Polin RA, Kirpalani H. Continuous positive airway pressure to prevent neonatal lung injury: how did we get here, and how do we improve? J Pediatr. 2016;173:17-24.e2. doi:10.1016/j.jpeds.2016.02.059 27025910

[16] Wu AW. Reaching common ground: the role of shared mental models in patient safety. J Patient Safety Risk Managet. 2018;23(5):183-184. doi:10.1177/2516043518805326

[17] Leotsakos A, Zheng H, Croteau R, . Standardization in patient safety: the WHO High 5s project. Int J Qual Health Care. 2014;26(2):109-116. doi:10.1093/intqhc/mzu010 24713313

[18] Braun D, Braun E, Chiu V, . Trends in neonatal intensive care unit utilization in a large integrated health care system. JAMA Netw Open. 2020;3(6):e205239. doi:10.1001/jamanetworkopen.2020.5239 32556257 PMC7303809

[19] Montgomery D. Introduction to Statistical Process Control. 4th ed. John Wiley & Sons; 2001.

[20] Schmitt SK, Sneed L, Phibbs CS. Costs of newborn care in California: a population-based study. Pediatrics. 2006;117(1):154-160. doi:10.1542/peds.2005-0484 16396873 PMC8720276

[21] Guillot M, Guo T, Ufkes S, . Mechanical ventilation duration, brainstem development, and neurodevelopment in children born preterm: a prospective cohort study. J Pediatr. 2020;S0022-3476(20)30653-3. doi:10.1016/j.jpeds.2020.05.039 32454115

[22] Furman L, Baley J, Borawski-Clark E, Aucott S, Hack M. Hospitalization as a measure of morbidity among very low birth weight infants with chronic lung disease. J Pediatr. 1996;128(4):447-452. doi:10.1016/S0022-3476(96)70353-0 8618176

[23] Schmidt B, Roberts RS, Davis PG, ; Caffeine for Apnea of Prematurity (CAP) Trial Investigators; Caffeine for Apnea of Prematurity CAP Trial Investigators. Prediction of late death or disability at age 5 years using a count of 3 neonatal morbidities in very low birth weight infants. J Pediatr. 2015;167(5):982-6.e2. doi:10.1016/j.jpeds.2015.07.067 26318030

[24] Klingenberg C, Wheeler KI, McCallion N, Morley CJ, Davis PG. Volume-targeted versus pressure-limited ventilation in neonates. Cochrane Database Syst Rev. 2017;10(10):CD003666. doi:10.1002/14651858.CD003666.pub4 29039883 PMC6485452

[25] Yonehara K, Ogawa R, Kamei Y, . Non-invasive neurally adjusted ventilatory assist versus nasal intermittent positive-pressure ventilation in preterm infants born before 30 weeks’ gestation. Pediatr Int. 2018;60(10):957-961. doi:10.1111/ped.13680 30133079

[26] Bahadue FL, Soll R. Early versus delayed selective surfactant treatment for neonatal respiratory distress syndrome. Cochrane Database Syst Rev. 2012;11(11):CD001456. doi:10.1002/14651858.CD001456.pub2 23152207 PMC7057030

[27] Isayama T, Chai-Adisaksopha C, McDonald SD. Noninvasive ventilation with vs without early surfactant to prevent chronic lung disease in preterm infants: a systematic review and meta-analysis. JAMA Pediatr. 2015;169(8):731-739. doi:10.1001/jamapediatrics.2015.0510 26053455

[28] Makker K, Cortez J, Jha K, . Comparison of extubation success using noninvasive positive pressure ventilation (NIPPV) versus noninvasive neurally adjusted ventilatory assist (NI-NAVA). J Perinatol. 2020;40(8):1202-1210. doi:10.1038/s41372-019-0578-4 31911641 PMC7222927

[29] Narchi H, Chedid F. Neurally adjusted ventilator assist in very low birth weight infants: current status. World J Methodol. 2015;5(2):62-67. doi:10.5662/wjm.v5.i2.62 26140273 PMC4482823

[30] Rosterman JL, Pallotto EK, Truog WE, . The impact of neurally adjusted ventilatory assist mode on respiratory severity score and energy expenditure in infants: a randomized crossover trial. J Perinatol. 2018;38(1):59-63. doi:10.1038/jp.2017.154 29072677

[31] Finer NN, Carlo WA, Walsh MC, ; SUPPORT Study Group of the Eunice Kennedy Shriver NICHD Neonatal Research Network. Early CPAP versus surfactant in extremely preterm infants. N Engl J Med. 2010;362(21):1970-1979. doi:10.1056/NEJMoa0911783 20472939 PMC3071534

[32] Schmidt B, Roberts RS, Davis P, ; Caffeine for Apnea of Prematurity Trial Group. Caffeine therapy for apnea of prematurity. N Engl J Med. 2006;354(20):2112-2121. doi:10.1056/NEJMoa054065 16707748

[33] Pakvasa MA, Saroha V, Patel RM. Optimizing caffeine use and risk of bronchopulmonary dysplasia in preterm infants: a systematic review, meta-analysis, and application of grading of recommendations assessment, development, and evaluation methodology. Clin Perinatol. 2018;45(2):273-291. doi:10.1016/j.clp.2018.01.012 29747888

[34] Schmidt B, Roberts RS, Davis P, ; Caffeine for Apnea of Prematurity Trial Group. Long-term effects of caffeine therapy for apnea of prematurity. N Engl J Med. 2007;357(19):1893-1902. doi:10.1056/NEJMoa073679 17989382

[35] Davis PG, Schmidt B, Roberts RS, ; Caffeine for Apnea of Prematurity Trial Group. Caffeine for Apnea of Prematurity trial: benefits may vary in subgroups. J Pediatr. 2010;156(3):382-387. doi:10.1016/j.jpeds.2009.09.069 19926098

[36] Araki S, Kato S, Namba F, Ota E. Vitamin A to prevent bronchopulmonary dysplasia in extremely low birth weight infants: a systematic review and meta-analysis. PLoS One. 2018;13(11):e0207730. doi:10.1371/journal.pone.0207730 30496228 PMC6264498

[37] Darlow BA, Graham PJ, Rojas-Reyes MX. Vitamin A supplementation to prevent mortality and short- and long-term morbidity in very low birth weight infants. Cochrane Database Syst Rev. 2016;2016(8):CD000501. doi:10.1002/14651858.CD000501.pub4 27552058 PMC7038719

[38] Tyson JE, Wright LL, Oh W, ; National Institute of Child Health and Human Development Neonatal Research Network. Vitamin A supplementation for extremely-low-birth-weight infants. N Engl J Med. 1999;340(25):1962-1968. doi:10.1056/NEJM199906243402505 10379020

[39] Nair V, Loganathan P, Soraisham AS. Azithromycin and other macrolides for prevention of bronchopulmonary dysplasia: a systematic review and meta-analysis. Neonatology. 2014;106(4):337-347. doi:10.1159/000363493 25278176

[40] Villamor-Martínez E, Pierro M, Cavallaro G, Mosca F, Kramer BW, Villamor E. Donor human milk protects against bronchopulmonary dysplasia: a systematic review and meta-analysis. Nutrients. 2018;10(2):238. doi:10.3390/nu10020238 29461479 PMC5852814

[41] Doyle LW, Cheong JL, Ehrenkranz RA, Halliday HL. Early (< 8 days) systemic postnatal corticosteroids for prevention of bronchopulmonary dysplasia in preterm infants. Cochrane Database Syst Rev. 2017;10(10):CD001146. doi:10.1002/14651858.CD001146.pub5 29063585 PMC6485683

[42] Filippone M, Nardo D, Bonadies L, Salvadori S, Baraldi E. Update on postnatal corticosteroids to prevent or treat bronchopulmonary dysplasia. Am J Perinatol. 2019;36(S 02):S58-S62. doi:10.1055/s-0039-1691802 31238361

[43] Shah SS, Ohlsson A, Halliday HL, Shah VS. Inhaled versus systemic corticosteroids for the treatment of bronchopulmonary dysplasia in ventilated very low birth weight preterm infants. Cochrane Database Syst Rev. 2017;10(10):CD002057. doi:10.1002/14651858.CD002057.pub4 29035425 PMC6485655

[44] Bassler D, Plavka R, Shinwell ES, ; NEUROSIS Trial Group. Early inhaled budesonide for the prevention of bronchopulmonary dysplasia. N Engl J Med. 2015;373(16):1497-1506. doi:10.1056/NEJMoa1501917 26465983

[45] Shaffer ML, Baud O, Lacaze-Masmonteil T, Peltoniemi OM, Bonsante F, Watterberg KL. Effect of prophylaxis for early adrenal insufficiency using low-dose hydrocortisone in very preterm infants: an individual patient data meta-analysis. J Pediatr. 2019;207:136-142.e5. doi:10.1016/j.jpeds.2018.10.004 30416014

[46] Darlow BA, Marschner SL, Donoghoe M, ; Benefits Of Oxygen Saturation Targeting-New Zealand (BOOST-NZ) Collaborative Group. Randomized controlled trial of oxygen saturation targets in very preterm infants: two year outcomes. J Pediatr. 2014;165(1):30-35.e2. doi:10.1016/j.jpeds.2014.01.017 24560181

[47] Drennan S, Szyld E. Should we target higher or lower oxygen saturation targets in the preterm infant? J Perinatol. 2019;39(5):758-760. doi:10.1038/s41372-019-0325-x 30692618

[48] Lam R, Schilling D, Scottoline B, . The effect of extended continuous positive airway pressure on changes in lung volumes in stable premature infants: a randomized controlled trial. J Pediatr. 2020;217:66-72. doi:10.1016/j.jpeds.2019.07.074 31519441 PMC7986570

[49] Manley BJ, Arnolda GRB, Wright IMR, ; HUNTER Trial Investigators. Nasal high-flow therapy for newborn infants in special care nurseries. N Engl J Med. 2019;380(21):2031-2040. doi:10.1056/NEJMoa1812077 31116919

[50] Mardegan V, Priante E, Lolli E, Lago P, Baraldi E. Heated, humidified high-flow nasal cannulae as a form of noninvasive respiratory support for preterm infants and children with acute respiratory failure. Am J Perinatol. 2016;33(11):1058-1061. doi:10.1055/s-0036-1586111 27603535

[51] McQueen M, Rojas J, Sun SC, . Safety and long term outcomes with high flow nasal cannula therapy in neonatology: a large retrospective cohort study. J Pulm Respir Med. 2014;4(6):216. doi:10.4172/2161-105X.1000216 26167395 PMC4497790

[52] Roberts CT, Owen LS, Manley BJ, ; HIPSTER Trial Investigators. Nasal high-flow therapy for primary respiratory support in preterm infants. N Engl J Med. 2016;375(12):1142-1151. doi:10.1056/NEJMoa1603694 27653564

[53] Ng G, da Silva O, Ohlsson A. Bronchodilators for the prevention and treatment of chronic lung disease in preterm infants. Cochrane Database Syst Rev. 2016;12(12):CD003214. doi:10.1002/14651858.CD003214.pub3 27960245 PMC6463958

[54] Thome UH, Genzel-Boroviczeny O, Bohnhorst B, ; PHELBI Study Group. Permissive hypercapnia in extremely low birthweight infants (PHELBI): a randomised controlled multicentre trial. Lancet Respir Med. 2015;3(7):534-543. doi:10.1016/S2213-2600(15)00204-0 26088180

[55] Stewart A, Brion LP. Intravenous or enteral loop diuretics for preterm infants with (or developing) chronic lung disease. Cochrane Database Syst Rev. 2011;2011(9):CD001453. doi:10.1002/14651858.CD001453.pub2 21901676 PMC7055198

[56] Ohlsson A, Aher SM. Early erythropoiesis-stimulating agents in preterm or low birth weight infants. Cochrane Database Syst Rev. 2020;2(2):CD004863. doi:10.1002/14651858.CD004863.pub6 32048730 PMC7014351

[57] Bui KCT, Kim R, Abbasi A, Nguyen M, Villosis MF, Chen Q. Erythropoietin treatment is associated with a reduction in moderate to severe bronchopulmonary dysplasia in preterm infants: a regional retrospective study. Early Hum Dev. 2019;137:104831. doi:10.1016/j.earlhumdev.2019.104831 31374455

[58] Juul SE, Comstock BA, Wadhawan R, ; PENUT Trial Consortium. A randomized trial of erythropoietin for neuroprotection in preterm infants. N Engl J Med. 2020;382(3):233-243. doi:10.1056/NEJMoa1907423 31940698 PMC7060076

[59] Rayjada N, Barton L, Chan LS, Plasencia S, Biniwale M, Bui KC. Decrease in incidence of bronchopulmonary dysplasia with erythropoietin administration in preterm infants: a retrospective study. Neonatology. 2012;102(4):287-292. doi:10.1159/000341615 22922736

[60] Oh W, Poindexter BB, Perritt R, ; Neonatal Research Network. Association between fluid intake and weight loss during the first ten days of life and risk of bronchopulmonary dysplasia in extremely low birth weight infants. J Pediatr. 2005;147(6):786-790. doi:10.1016/j.jpeds.2005.06.039 16356432

[61] Barrington KJ, Fortin-Pellerin E, Pennaforte T. Fluid restriction for treatment of preterm infants with chronic lung disease. Cochrane Database Syst Rev. 2017;2(2):CD005389. doi:10.1002/14651858.CD005389.pub2 28176308 PMC6464249

[62] Sankar MN, Bhombal S, Benitz WE. PDA: to treat or not to treat. Congenit Heart Dis. 2019;14(1):46-51. doi:10.1111/chd.12708 30811796

[63] Clyman RI, Liebowitz M. Treatment and nontreatment of the patent ductus arteriosus: identifying their roles in neonatal morbidity. J Pediatr. 2017;189:13-17. doi:10.1016/j.jpeds.2017.06.054 28709633 PMC5639904

[64] Hagadorn JI, Bennett MV, Brownell EA, Payton KSE, Benitz WE, Lee HC. Covariation of neonatal intensive care unit-level patent ductus arteriosus management and in-neonatal intensive care unit outcomes following preterm birth. J Pediatr. 2018;203:225-233.e1. doi:10.1016/j.jpeds.2018.07.025 30243544

[65] Ting JY, Roberts A, Sherlock R, ; Canadian Neonatal Network Investigators. Duration of initial empirical antibiotic therapy and outcomes in very low birth weight infants. Pediatrics. 2019;143(3):e20182286. doi:10.1542/peds.2018-2286 30819968

[66] Lavelle J, Schast A, Keren R. Standardizing care processes and improving quality using pathways and continuous quality improvement. Curr Treatment Options Pediatr. 2015;1(4):347-358. doi:10.1007/s40746-015-0026-4

[67] Sant’Anna GM, Keszler M. Developing a neonatal unit ventilation protocol for the preterm baby. Early Hum Dev. 2012;88(12):925-929. doi:10.1016/j.earlhumdev.2012.09.010 23058298

