# Supplementary material for: Rates of Bronchopulmonary Dysplasia Following Implementation of a Novel Prevention Bundle
Source: JAMA Netw Open. 2021 Jun 28;4(6):e2114140. doi: 10.1001/jamanetworkopen.2021.14140 (PMC8239950; doi:10.1001/jamanetworkopen.2021.14140)
Supplement: Supplement. — eAppendix 1. Shrunken Standardized Morbidity Ratio eFigure 1. No BPD Road Map: Ventilatory Management for High-Risk Infants of GA <28 Weeks or BW <1000 g eFigure 2. No BPD Road Map: Ventilatory Management for High-Risk Infants of GA 28-31 Weeks and BW ≥1000 g eFigure 3. No BPD Road Map: Nonventilatory Management/Pharmacologic Therapies and Other Strategies for Very Low-Birth-Weight Infants (BW <1500 g) eFigure 4. Risk-Adjusted Mortality in Infants 501 to 1500 g eFigure 5. Risk-Adjusted Mortality or Specified Morbidities in Infants 501 to 1500 g eAppendix 2. BPD Prevention Bundle Implementation [file jamanetwopen-e2114140-s001.pdf]

Supplementary Online Content

Villosis MFB, Barseghyan K, Ambat MT, Rezaie KK, Braun D. Rates of bronchopulmonary dysplasia following implementation of a novel prevention bundle. *JAMA Netw Open*. 2021;4(6):e2114140. doi:10.1001/jamanetworkopen.2021.14140

eAppendix 1. Shrunken Standardized Morbidity Ratio
eFigure 1. No BPD Road Map: Ventilatory Management for High-Risk Infants of GA <28 Weeks or BW <1000 g
eFigure 2. No BPD Road Map: Ventilatory Management for High-Risk Infants of GA 28-31 Weeks and BW ≥1000 g
eFigure 3. No BPD Road Map: Nonventilatory Management/Pharmacologic Therapies and Other Strategies for Very Low-Birth-Weight Infants (BW <1500 g)
eFigure 4. Risk-Adjusted Mortality in Infants 501 to 1500 g
eFigure 5. Risk-Adjusted Mortality or Specified Morbidities in Infants 501 to 1500 g
eAppendix 2. BPD Prevention Bundle Implementation

This supplementary material has been provided by the authors to give readers additional information about their work.

© 2021 Villosis MFB et al. *JAMA Network Open*.

eAppendix 1. Shrunken Standardized Morbidity Ratio (SMR)

"Shrunken standardized morbidity ratio" (SMR) is the term Vermont Oxford uses to describe the risk-adjusted outcomes they produce from their registry. They provide these outcomes for a large range of measures annually to each member NICU. The calculation of the shrunken standardized morbidity ratio is described in Horbar JD, et al. Variation in performance of neonatal intensive care units in the United States. JAMA Pediatr 2017;171(3): e164396. Paraphrasing the article, empirical Bayes shrinkage estimators are used to compute standardized morbidity ratios for each hospital using a hierarchical logistic regression model that assumes a posterior gamma Poisson distribution. The comparison data are the patient level data for patients born in the same year at the approximately 750 Vermont Oxford member NICUs in the United States. These centers care for about 90% of all very low birth weight babies in this country.

The SMR includes patient-level adjustments for selected risk factors: gestational age, birth weight, SGA (<=10 percentile), severity of birth defect, multiple gestation, Apgar score at 1 minute, infant sex, vaginal delivery, birth location (inborn/outborn), altitude of center.

The Vermont Oxford SMRs presented in the article were for BPD<33, mortality and "mortality or selected morbidities". Specifically, (1) BPD<33 analyzes the rate of babies with birth weights from 501-1500 g with GA <33 wk at birth who survive to 36 weeks' postmenstrual age and who are on supplemental oxygen on that day or, if discharged before 36 weeks, on the day of discharge. (2) Mortality analyzes the rate of death before final NICU discharge among babies born at the center or admitted to the center's NICU with birth weights from 501-1500 g, (3) "mortality or specified morbidity" analyzes the rate of babies with any of the following outcomes: mortality before home discharge, pneumothorax, Chronic lung disease or BPD (BPD<33), stage 2 or higher necrotizing enterocolitis (NEC), nosocomial infection, severe periventricular-intraventricular hemorrhage (IVH), and/or cystic periventricular leukomalacia. From 1995 to 2010, Extreme length of stay (LOS) was included as part of the definition. From 1995 to 2013 BPD was not limited to BPD<33. From 2013 it was changed to BPD<33.

© 2021 Villosis MFB et al. *JAMA Network Open*.

eFigure 1. No BPD Road Map: Ventilatory Management for High-Risk Infants of GA <28 Weeks or BW <1000 g

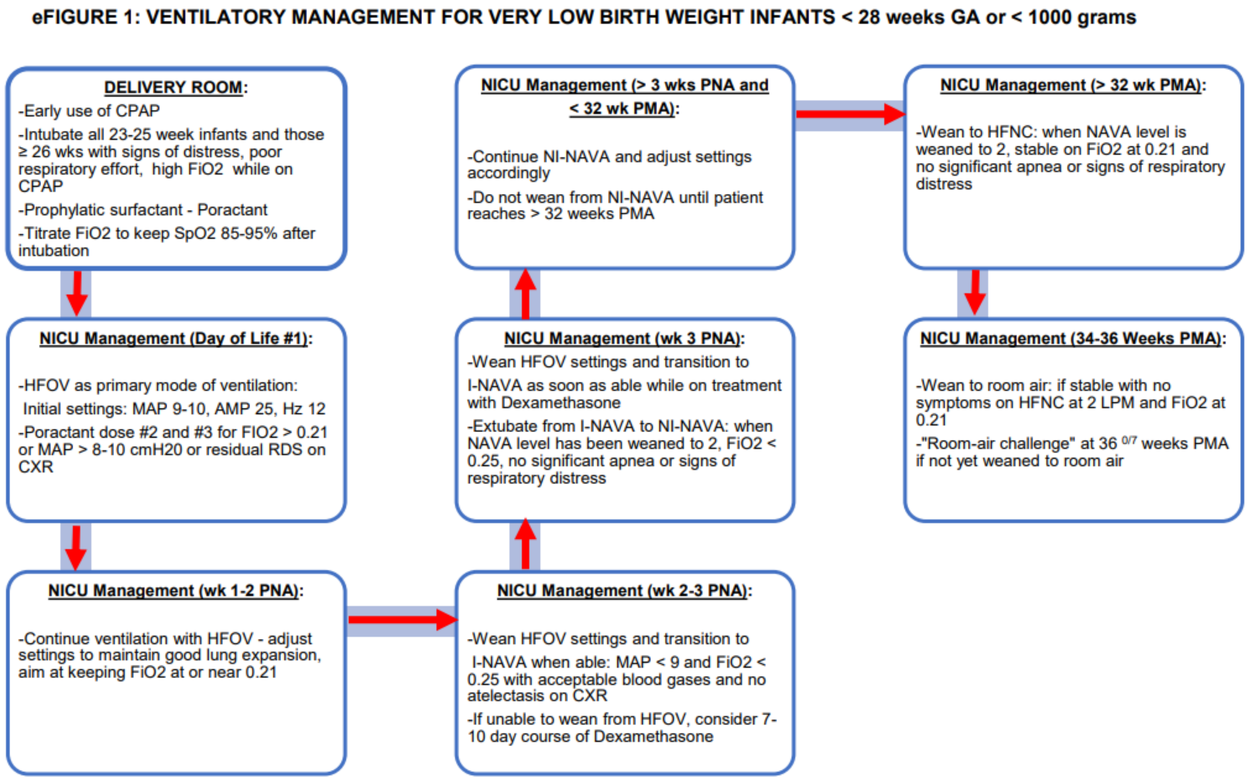

HFV: high frequency oscillatory ventilation. AMP: amplitude. DR: delivery room. CPAP: continuous positive airway pressure. CXR: chest radiograph. I-NAVA. Endotracheal neurally adjusted ventilatory assist. MAP: mean airway pressure. NI-NAVA: nasal neurally adjusted ventilatory assist. PMA: postmenstrual age. PNA: postnatal age. RDS: respiratory distress syndrome.

© 2021 Villosis MFB et al. *JAMA Network Open*.

eFigure 2. No BPD Road Map: Ventilatory Management for High-Risk Infants of GA 28-31 Weeks and BW ≥1000 g

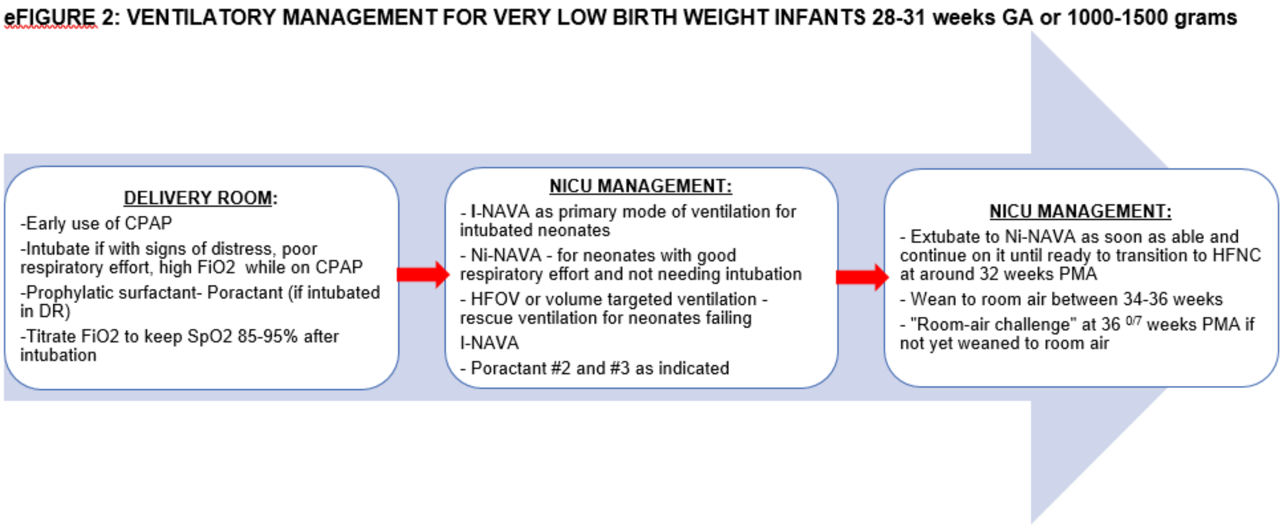

HFV: high frequency oscillatory ventilation. AMP: amplitude. DR: delivery room. CPAP: continuous positive airway pressure. CXR: chest radiograph. I-NAVA. Endotracheal neurally adjusted ventilatory assist. MAP: mean airway pressure. NI-NAVA: nasal neurally adjusted ventilatory assist. PMA: postmenstrual age. PNA: postnatal age. RDS: respiratory distress syndrome.

© 2021 Villosis MFB et al. *JAMA Network Open*.

eFigure 3. No BPD Road Map: Nonventilatory Management/Pharmacologic Therapies and Other Strategies for Very Low-Birth-Weight Infants (BW <1500 g)

eFIGURE 3: PHARMACOLOGIC THERAPIES AND OTHER STRATEGIES on our ROAD to NO BPD for VERY LOW BIRTH WEIGHT INFANTS (BW < 1500 grams)

- ☐ Early or prophylactic use of surfactant within 15 minutes of birth and subsequent doses using clinical and radiologic parameters during weeks 1-2
- ☐ Starting caffeine on postnatal day 1 instead of later
- ☐ Subcutaneous erythropoietin analog starting in the 2nd week of life until discharge from the NICU
- ☐ Antibiotic stewardship and infection prevention efforts including CLABSI and VAP prevention bundles and high threshold for starting antibiotics in the setting of respiratory decompensation
- ☐ Conservative treatment approach of Patent Ductus Arteriosus – non-intervention strategy and close observation with serial echocardiograms
- ☐ Fluid restriction balanced by provision of complete nutrient intake
- ☐ Avoiding the use of diuretics, antacids and anti-reflux medications
- ☐ Promoting optimal nutrition by following a strict feeding protocol; high usage of breast milk to achieve adequate growth and repair
- ☐ Neurodevelopmental assistance - early OT referral, minimization of pain and stress, containment during unavoidable painful and/or stressful stimuli, avoidance or decreased use of neurotropic medications such as narcotics or sedatives
- ☐ Family-centered care approach by encouraging parental involvement
- ☐ Multi-disciplinary team approach

Additional Treatments for Higher Risk Infants (< 28 weeks or < 1000 grams)

- ☐ Vitamin A intramuscularly for the first four postnatal weeks
- ☐ Inhaled beta2-agonist and inhaled steroids starting on day 2-3 until discontinuation of respiratory support
- ☐ Intravenous dexamethasone for infants requiring mechanical ventilation at the end of their second postnatal week
- ☐ Screening for ureaplasma/mycoplasma in tracheal aspirate soon after birth and treatment with azithromycin until the culture results come back

© 2021 Villosis MFB et al. *JAMA Network Open*.

eFigure 4. Risk-Adjusted Mortality in Infants 501 to 1500 g

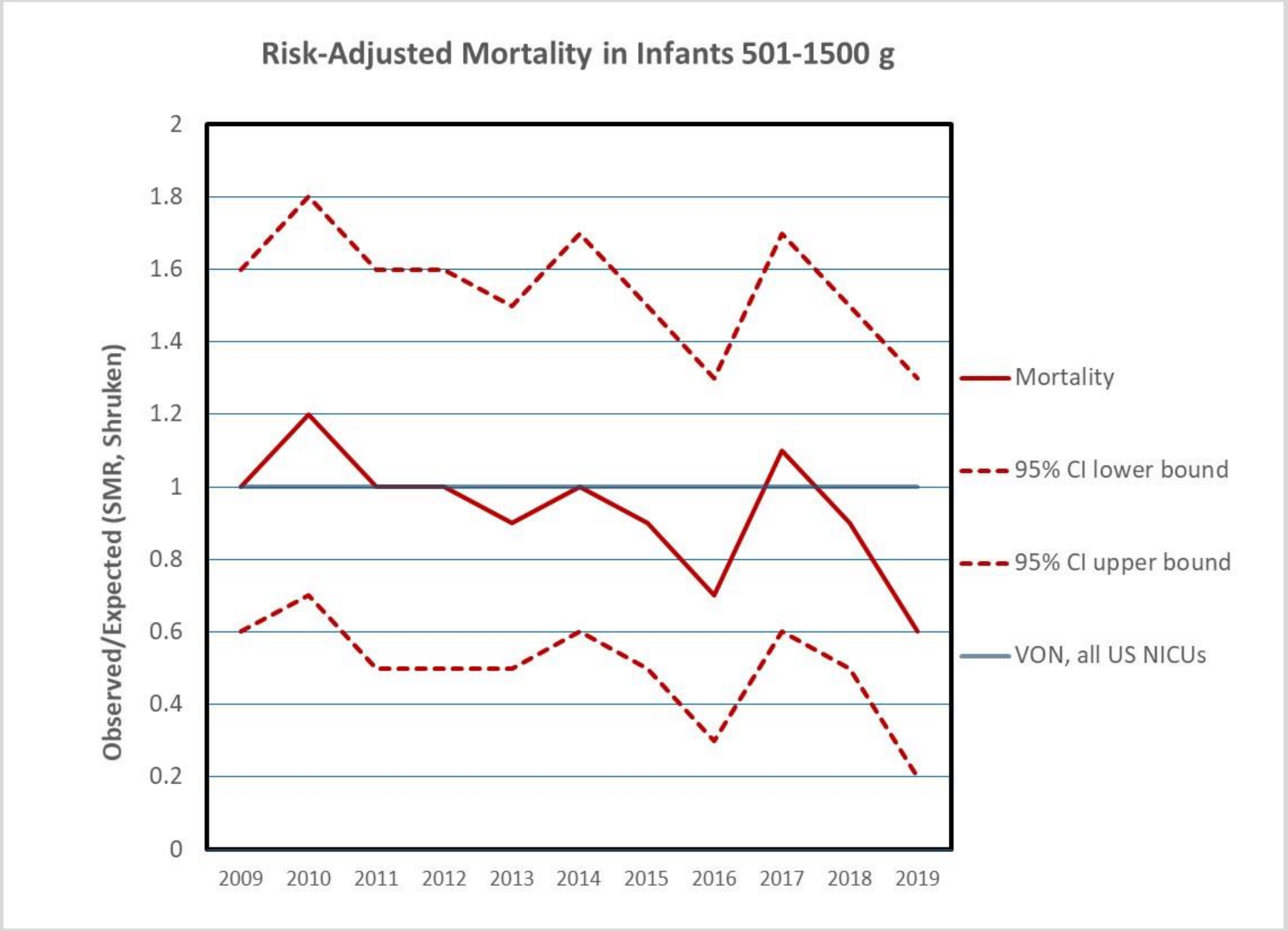

CI: confidence interval. NICU: neonatal intensive care unit. SMR: standardized morbidity ratio. VON: Vermont Oxford Network. US: United States. Solid red line: Mortality SMR, shrunken. Solid blue line: VON, all US NICUs. Dashed red line: 95% CI lower or upper bound.

© 2021 Villosis MFB et al. *JAMA Network Open*.

eFigure 5. Risk-Adjusted Mortality or Specified Morbidities in Infants 501 to 1500 g

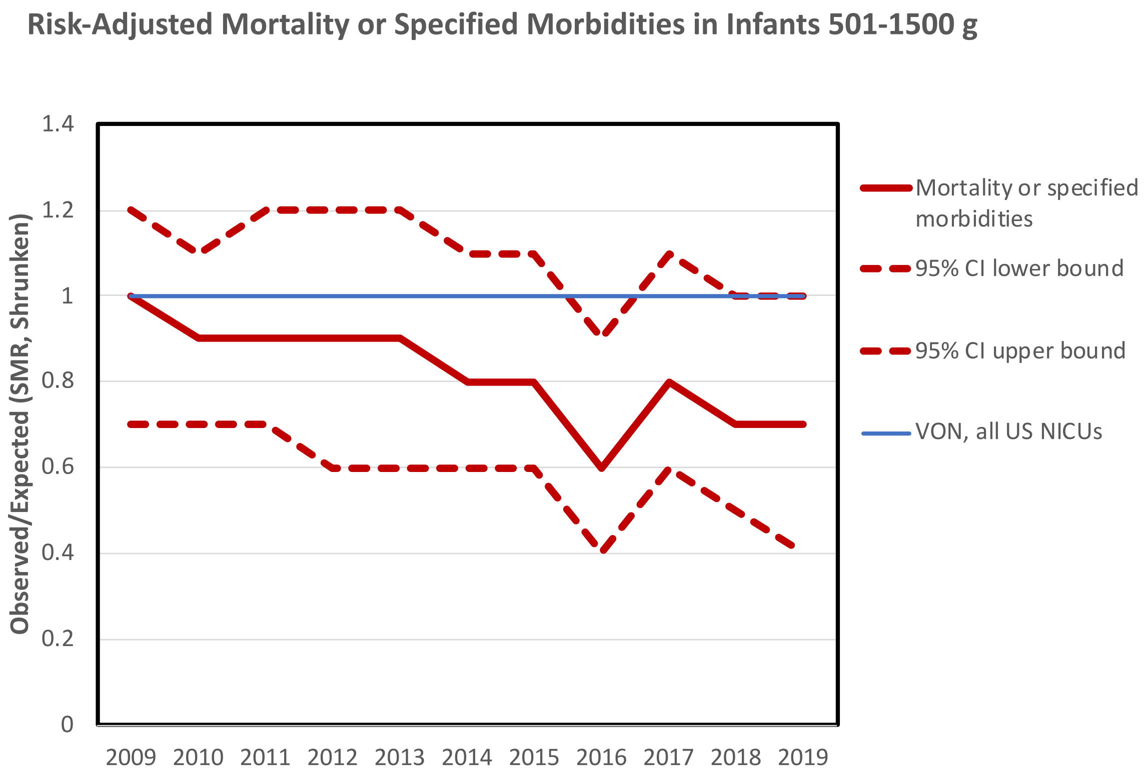

CI confidence interval. NICU: neonatal intensive care unit. SMR: standardized morbidity ratio. VON: Vermont Oxford Network. US: United States. Solid red line: Mortality or specified morbidities SMR, shrunken. Solid blue line: VON, all US NICUs. Dashed red line: 95% CI lower or upper bound.

© 2021 Villosis MFB et al. *JAMA Network Open*.

eAppendix 2. BPD Prevention Bundle Implementation

Ventilatory support:
Delivery room (DR) management (early CPAP, intubation): All infants ≤ 32 weeks gestation (GA) were started on CPAP immediately after birth by using a T-piece resuscitator with fraction of inspired oxygen (FiO2) at 0.21-0.30 and titrated to meet the Neonatal Resuscitation Program (NRP) saturation goals. Positive pressure ventilation (PPV) was provided per the NRP guidelines if needed. All infants born at GA of 23-25 weeks were intubated in the DR while older premature infants (≥ 26 weeks GA) were intubated in the DR or NICU for surfactant therapy if they showed signs of significant respiratory distress, poor respiratory effort and/or were requiring FiO2 ≥ 0.30 at CPAP level of 6 cmH2O. We initially used early trial of CPAP to manage respiratory distress syndrome (RDS) but had a lower threshold for intubation. We opted to intubate all infants born at less than 26 weeks GA.

High frequency oscillatory ventilation (HFV) was delivered using the Sensormedics 3100A ventilator (Carefusion, Yorba Linda, CA). HFV was predominantly used as a rescue ventilation strategy in the initial changes period. Criteria for rescue were MAP ≥ 10 and FIO2 > 0.25-0.30. In the full implementation period, we used HFV as the primary mode of ventilation for management of RDS in high risk intubated infants (< 28 weeks GA and/or birth weight (BW) < 1000 g) with starting settings of MAP 9-10 Hz 12 Amp 25. Criteria for transitioning to lower acuity respiratory care were a MAP of about < 9, FIO2 < 0.25, acceptable blood gases, and no atelectasis on CXR. In full implementation period, for GA < 28 weeks and BW < 1000g infants were also kept on HFV for at least one week. Infants were generally weaned from HFV to neurally adjusted ventilatory assist (NAVA).
Volume-targeted ventilation was delivered using Drager Babylog 8000 or VN500 (Drager US. Telford, PA). It was the primary mode of invasive ventilation used for all infants in the initial changes period and for GA > 28 weeks and BW > 1000 g in the full implementation period. We used the PC-PSV-VG mode with PIP max 30 PEEP 6, ITmax 0.4, backup rate 40, Volume 4-6 mL/kg. Criteria for extubation were a MAP of about 8 cmH20 while meeting FIO2/work of breathing/CXR/blood gas monitoring targets. In the initial changes period infants were extubated from volume ventilation to CPAP or NIMV. In the full implementation period, infants, especially in the < 28 weeks GA group, were generally extubated to non-invasive (NI) NAVA.

Invasive NAVA (I-NAVA): I-NAVA was delivered using Maquet Servo-I ventilator (Maquet Medical Systems USA, Wayne NJ). Initial settings were NAVA level 6, Pmax 15-18 above PEEP, PEEP 6, backup rate 40, backup IT 0.4. I-NAVA was usually a phase that lasted hours to days rather than weeks. Criteria for extubation were at least four hours of meeting FIO2/work of breathing/CXR/blood gas monitoring targets. If the "% backup" rate was more than about 30%, infants were sometimes tested on a lower backup rate to confirm adequate respiratory drive before extubation.

NI-NAVA: We started using the RAM cannula (Neotech, Valencia, CA) in 2012 as the nasal interface. Initial settings were NAVA level 4-6, Pmax 15-18 above PEEP, PEEP 6, backup rate 40, backup IT 0.4. In the initial changes period, criteria for weaning to CPAP or NIMV were hours to days of meeting FIO2/work of breathing/CXR/blood gas monitoring targets. In the full implementation period infants were kept on NI- NAVA until at least 32 weeks PMA.

High flow nasal cannula (HFNC): VLBW infants were maintained on NI-NAVA until criteria to transition to HFNC were fulfilled which occurred at around 32 weeks PMA at the earliest. For the majority of the full implementation period, we used the Vapotherm Precision Flow (Exeter, NH) initiated by the end of 2014 for HFNC delivery. We used HFNC as a transitional modality to wean off respiratory support before 36 weeks PMA but not as a primary respiratory support for RDS.

Oxygen saturation targets: During invasive and non-invasive ventilation, we kept our saturation targets between 85% to 95% while on supplemental oxygen and >=85% when on FIO2 0.21 until 34 weeks PMA, after which the saturation goal was >=94%.

FIO2/work of breathing management: During invasive and non-invasive ventilation, respiratory settings were adjusted so that each day the FIO2 reached 0.21 without retractions or desaturations.

© 2021 Villosis MFB et al. *JAMA Network Open*.

CXR and blood gas monitoring: CXR and blood gas were routinely obtained on admission. Thereafter, CXR was taken daily while on HFV and blood gas every 4-6 hours during the first few hours of admission or more frequently as needed then reduced to q 12 hourly once stabilized. Additionally, CXR and blood gas were obtained on as needed basis for various clinical indications such as increased desaturations and FIO2 requirement and or worsening work of breathing. Respiratory settings were adjusted to keep lungs inflated to about 9-10th ribs with relatively clear lung fields and to maintain pH and partial pressure of carbon dioxide (pCO_2) levels within normal ranges (35-45 mmHg up to low 50s). Targeting high pCO_2, a strategy used in permissive hypercarbia, was not used in our NICU.

High expectations were adopted for acceptable respiratory status and focus on full alveolar recruitment. For example, FiO2 > 0.21 was itself considered an actionable finding. Focused respiratory care management was central. Patent ductus arteriosus (PDA), pneumonia, or reflux were rarely accepted as reasons for respiratory deterioration, minimizing the diversion of our attention from efforts to prevent or treat alveolar de-recruitment and pulmonary effects of the immature dysregulated immune system. Examples of PMA-specific practices different from common practices were, for GA < 28 weeks, prophylactic HFV and transition to invasive NAVA followed by extubation directly to non-invasive NAVA until at least 32 weeks PMA. The system of care in the full implementation period is described in "No BPD Roadmap – Ventilatory Management" (eFigure 1 and 2).

Pharmacologic Respiratory Therapies:
Surfactant: In our unit, surfactant was administered early and prophylactically via a thin intra-tracheal catheter within the first 15 minutes after birth if intubated in the DR and as a rescue surfactant if intubated in the NICU. In full implementation period, more infants received surfactant in the DR. Subsequent doses were given during weeks 1-2 if the infants remained intubated using clinical (e.g. FiO_2 > 0.25, MAP >10) and radiologic parameters.

Caffeine: We administered Caffeine to infants less than 32 weeks PMA from day one and at the onset of recurrent and significant apnea in older premature infants (20 mg/kg loading, 5-10 mg/kg/day maintenance dosing of caffeine citrate). Treatment was discontinued at 34 weeks PMA if there were no symptoms of apnea of prematurity, if the infant was off respiratory support, or two days after discontinuation of respiratory support, whichever came later. Subsequently, if cardiorespiratory events requiring stimulation occurred, caffeine was restarted, and the infant was discharged home on caffeine after having five consecutive days without cardiorespiratory events that required stimulation.

Inhaled therapies: The use of budesonide and albuterol was incorporated in our "BPD prevention bundle". Both treatments were started by day 3 and continued until termination of any type of respiratory support. In initial changes period, these treatments were used rather sporadically and inconsistently. Inhaled corticosteroids, due to their anti-inflammatory properties, may offer beneficial effects on the pulmonary system with a lower risk of undesirable systemic side effects.

Systemic steroids: Despite a slight tendency for higher use of postnatal systemic steroids in full implementation period, our utilization rate was similar between the two study periods with difference in the timing of the initiation of the treatment. In initial changes period, the treatment was initiated as rescue therapy when infants remained intubated at around 3-4 weeks of age. In full implementation period, infants who continued to require mechanical ventilation (usually HFV with MAP > 9) at the end of their second postnatal week and were remote from being extubated to NI-NAVA, were treated with a tapering course of intravenous dexamethasone given over 7-10 days. We have not used hydrocortisone for BPD prevention or treatment in our unit.

Vitamin A: We used Vitamin A during both study periods for infants < 1000 g or < 28 weeks except when it was on national shortage in 2012-2014.

We rarely used sedatives during ventilation. We considered agitation primarily a sign of air hunger or discomfort and responded to agitation by trials of increasing respiratory support or improving comfort with nonpharmacological interventions such as adjusting positioning and bundling of the infant.

Antibiotic stewardship:

Empiric antibiotics were started on admission if symptoms and risk factors for sepsis were present and were stopped at 36-48 hours of therapy if blood cultures remained negative. We also maintained a high threshold for restarting antibiotics for any form of respiratory decompensation unless sepsis was highly suspected due to the presence of other systemic signs or laboratory findings. Due to historically high prevalence of mycoplasma/ureaplasma in our patient population, high risk intubated infants were treated with intravenous azithromycin upon admission to the NICU while awaiting culture results.

IVIG:
Intravenous immunoglobulin (IVIG) was used once a week for infants born less than 1250 grams until they reached 1250 grams.

Nutrition:
Optimal nutrition is considered cornerstone in the prevention and treatment of BPD. The feeding guideline used in our unit is a human milk-based guideline that utilizes mother's breastmilk or donor breastmilk as needed depending on mother's ability to provide adequate supply of breastmilk for her infant. Minimal Enteral Nutrition (MEN) and probiotics were started as soon as clinically permitted, with the goal of initiating the first feeding and the first dose of probiotics within the first 12-24 hours after birth. After seven days of MEN, enteral feedings were advanced daily by 20 ml/kg/day as tolerated. Total parenteral nutrition (TPN) and peripherally inserted central catheter (PICC) were discontinued on or before the end of the second week when enteral nutrition had reached 120 mL/kg/day. We encouraged and provided support to our mothers to sustain their breast milk production. Our efforts resulted in higher usage of human milk at discharge for our infants.

In terms of parenteral nutrition, amino acids were started soon after birth using "starter TPN" and the dose was maximized in standard TPN up to 4-4.5 grams/kg/day. Intralipids were initiated on day 1-2 and maximized to 2.5 grams/kg/day over the course of 2-3 days. The caloric goal was 100 kcal/kg/day if given intravenously by TPN and 120-150 kcal/kg/day for enteral feeds. Insulin drip was utilized to optimize non-protein calorie energy and glucose intake in infants with persistent hyperglycemia defined as blood glucose level greater than 160 mg/dL on three consecutive measurements.

We used modest fluid restriction with very infrequent use to almost complete avoidance of diuretics to manage impairment of lung mechanics presumably from associated increased lung water content. The balance between fluid restriction and provision of complete nutrient intake (using fortifiers and creams as caloric enhancers) to achieve adequate growth was key to our fluid and nutritional management. Fluids were restricted at 120 ml/kg/day with consideration to liberalization of the fluids during the first week depending on the degree of weight loss and hypernatremia. Enteral feeds were gradually advanced to 150 ml/kg/day when the ductus was closed and/or the infant was weaned off from respiratory support.

PDA management
In full implementation period, we used conservative management for PDA which is a non-intervention strategy with close clinical observation, serial echocardiograms and avoidance of any pharmacologic treatment or ligation following very strict criteria. Treatment of moderate to large PDA, either pharmacologically or surgically, was not indicated solely due to continued invasive mechanical ventilation or respiratory decompensation during weeks 1-3. With this treatment approach, we found high spontaneous PDA closure rates without increase in adverse outcomes.

Hematologic:
We strived to minimize unnecessary blood draws, iatrogenic anemia, and blood transfusions. During the study periods, infants less than 32 weeks GA and/or with BW less than 1500 grams were generally started on recombinant human erythropoietin (rh EPO), Ferrous Sulfate and Vitamin E supplementation when they reached 100 mL/kg/day of enteral feeds, given thrice weekly until discharge.

Nursing-specific care
Nursing-specific care included early occupational therapy in the unit, minimization of pain and stress by clustering care and procedures, containment during unavoidable painful and/or stressful stimuli, comforting modalities such as kangaroo care/human touch, fluidized positioners and music, and more importantly, encouragement of family presence and involvement.

© 2021 Villosis MFB et al. *JAMA Network Open*.
